# An empirical evaluation of stakeholders perceptions towards coach education in Oman: effectiveness, barriers, and privatization

**DOI:** 10.3389/fspor.2026.1802694

**Published:** 2026-06-18

**Authors:** Majid AL Busafi, Nabil Gmada, Ali Al-Yaaribi, Asharul Khan

**Affiliations:** 1 Department of Physical Education & Sports Sciences, College of Education, Humanities Research Center, Sultan Qaboos University, Muscat, Oman; 2 Humanities Research Center, Sultan Qaboos University, Muscat, Oman

**Keywords:** barriers, coach education, effectiveness, empirical evaluation, Oman, policy, privatization, sports development

## Abstract

**Purpose:**

This research critically examines how adequately currently available coach education programs supports sports coaching and sports development in Oman. It also investigates how the perceptions towards coach education effectiveness, barriers, and privatization are influenced by variables such as job title and experience.

**Methodology:**

An exploratory cross-sectional case study design and quantitative measures have been used to investigate coach education programs in Oman. A total of 159 stakeholders provided responses through a nation-wide online survey that was administered from September 10 to November 15, 2024. Data collected in this study were analyzed using a number of techniques, including the Kruskal–Wallis *H* test and the Mann–Whitney *U*-test.

**Findings:**

The finding indicates that the current coach education program in Oman is underdeveloped and ineffective. More specifically 75.7% of sports stakeholders support reform in coach education. The stakeholders exhibited modest differences towards coach education privatization. While privatization provides opportunities for flexibility and revenue-generation, it is not a sufficient answer on its own due to trade-offs inherent to equity, accountability, and sustainability. The way forward would involve creating a multitude of funding sources with strong regulation so as to ensure compliance across the system. Further, the findings suggest that a hybrid system (i.e., combining public oversight with private and digital innovations) may represent a promising approach to addressing existing challenges in coach education. Finally, improving coach education in Oman necessitates systemic reform through the better integration of governance, pedagogy, and contextual relevance.

## 1 Introduction

Coach education has been widely identified as a process to provide sports coaches with both theoretical and practical knowledge. The systematic review by Li et al. (1) found that coach education positively impacts coaching effectiveness. The existing scholarships identifies coach education as having an impact on setting professional standards and improving the performance of athletes (2–4), but most of the literature assumes a direct or linear relationship exists between formal education and the quality of coaching. This is a problematic assumption because there is a large body of research that consistently demonstrates that obtaining a formal certification does not always lead to improvements in coaching methods or athlete outcomes. According to Trudel et al. (5) an assessment of coach education can help in understanding the challenges.

In recent years, the use of technocratic and competency-based models has resulted in a dominant framing of coaching in terms of learning to coach as a skills set. While such models have been effective for many athletes and other professions, they do not capture the intricacies of coaching as being an adaptive, relational, and contextual practice. Additionally, as sports become more professionalized and commercialized, coaches are now responsible for fulfilling many different roles, often simultaneously. These include educator, mentor, psychologist, strategist, leader, and organizational manager (6). Although the coach's role has changed to include elements beyond simply providing technical instruction, many coaching education programs continue to focus on providing technical or tactical skill acquisition through narrowly designed curriculum. Mason et al. (7), point out that while coaching involves aspects of both “art” and “science,” many of the formalized systems that are available primarily focus on providing standardized and scientific types of knowledge and do not place enough value on experiential and reflective types of learning.

The introduction of university-based degree programs for coaching reflects the growing importance of coach education. The maturation of undergraduate coach programs in the USA (8), and frameworks (e.g., International Sport Coaching Framework (ISCF), European Coaching Framework) reflects an attempt to professionalize coaching in the western world (9). Nonetheless, the professionalization agenda should remain subject to critical examination. Many standardized coach education frameworks and certification function under the assumption of universal applicability and may not adequately reflect the realities of coaching practices (10, 11) due to variations in governance structures, expectations, and strategic priorities (12–14). In order for coaches to be effectively prepared to work with athletes from diverse cultures, they need to learn through experience and context rather than solely through didactic instruction (15). This presents another contradiction in existing coach education systems.

Furthermore, scholars including (16–18) criticized traditional “train and certify” methods for treating the coach as an audience participant, rather than as a learner who is actively creating an understanding through experience. Many challenges remain in coach education, with barriers to implementation even in effective programs (3, 19). Sports coaches need to integrate technical knowledge with professional responsibility, interpersonal skills, and personal qualities to be effective, as highlighted by (20) and supported by (21). Therefore, it is likely that a major obstacle to a coach's success in coaching is not simply access to education, but rather the epistemological approaches that create and validate a coach's knowledge. These structural weaknesses are of major importance in terms of the larger context of neoliberal reform of education and professional training.

According to (22) the education systems, (i.e., coaching, professional development), have been consistently re-engineered to respond more effectively and creatively to these demands. The privatization of educational systems is presented as a means to solve the ineffectiveness of public systems through increased innovation, flexibility, and responsiveness to market needs. Santalova and Põder (23) point out the inherent trade-offs that exist between efficiency and equity within privatized education systems and call into question whether privatization will lead to greater inequality, fragmentation of governance, and prioritization of commercial interest at the expense of development outcomes. The risks associated with these trends are heightened in coach education.

The empirical study on how these tensions materialise in coach education systems is currently lacking, particularly in non-western nations. The existing research on coach education in Oman demonstrates a fragmented and undeveloped coach education system that completely rely on government funding. While governmental monopolisation has provided some stability in structure, it continues to inhibit innovation, responsiveness and variation within coach development pathways. Privatization continues to receive some consideration as a potential reform, but the discourse surrounding this potential method of restructuring is somewhat speculative, with very few voices of stakeholders contributing to these discussions.

Thus, the critical issue is not whether privatization is an inevitable reform but the critical question remains: Are current coach education structures capable of meeting sport's needs? If the current coach education systems cannot accommodate sports developmental needs, then what kind of reforms are required? At this point, there is neither empirical knowledge on how the coach education system is viewed by coaches, athletes and sport administrators nor there is quantitative evidence to indicate whether the adoption of a privatised coach education system would resolve current inadequacies without imposing new inequities on the coach education system. Therefore, this study seeks to address this gap by critically examining how adequately the currently available coach education program supports sports coaching and sports development in Oman.

The structure of the paper is as follows: Section (2) contains an overview of the literature relevant to the research topic; Section (3) describes the research methods that were utilized; Section (4) details the data analysis; Section (5) discusses the implications of the findings from the data analysis (include lacking detail), and Section (6) contains a summary of the key findings and final conclusions.

## 2 Literature review

Coaching athletes is a complex, multifaceted profession that consists of teaching, leading, and developing an athlete's holistic potential. While there is general agreement among researchers that coach education lies at the core of an effective coach, few, if any, published literature have raised critical questions regarding challenges and solutions on coaching and coach education for the better outcomes from athletes in the developing countries.

### 2.1 Coach education

A number of studies (experimental and quasi-experimental) of coach/mentor programs support the notion that coaches have improved coaching performance when receiving mentoring. Coach education programs were found in a large, contemporary meta-analysis (1) to have moderate to large positive effects on effectiveness of coaching capabilities and on both physical and psychological outcomes for youth athletes. The coach/athlete relationship has been strengthened and task focus increased — variables that have been consistently found to be linked to positive athlete development and competitive outcomes (24–26).

Studies have shown coach education is still missing important components of effective design. This has resulted in an overall perception that the courses are disconnected, top-down oriented, and lack real-world application (27, 28, 77). Therefore, coaches prefer ways of learning that are informal and social; however, they do not have the resources available to engage effectively in the experiential aspect of this form of learning (29, 30). Formal coaching education is often viewed as costly, rigid, and low-impact, where much of its nature is dictated by market-oriented logics or governing bodies (28, 31, 32). Furthermore, these educational systems tend to create standardised/competence-based learning models that provide technically effective but critically underdeveloped practitioners who fit into (`neo-liberal’) performance-oriented sport cultures (27, 33, 78).

Moreover, studies have indicated that a successful coach education program should place emphasis on contextualized, problem-based learning, as well as providing continued mentoring with structured opportunities to implement new learning in practice settings (31, 34, 35). These design principles are in stark contrast to the decontextualized, prescriptive, and theory-based design that have received considerable criticism in the literature. In particular, the use of democratic, dialogic approaches in which co-constructing knowledge with a “more knowledgeable other,” such as an experienced mentor or knowledgeable facilitator is believed to support positive and sustained change in coaching practice (36, 37).

### 2.2 Privatization of education

The privatization of education comprises multiple policy trajectories (competing reforms) that vary by context and can have differing impacts on equity, governance, and professional development. A nuanced look at published research on this topic supports it. For example, providing choice, improving efficiency, and in some cases brings innovation. On the other hand privatization also impedes access (through race and socio-economic status), limiting power within communities, and transforming knowledge production processes. Thus it raises significant questions about if/how education and coach education are considered to be public goods.

#### 2.2.1 Variegated privatization

The human capital and market perspectives provide the primary rationale for privatization. Education is conceptualized as a tradable commodity such that competition between providers is expected to promote quality and efficiency improvement (38, 39). The human capital theory supports this concept by associating productivity and economic growth with education, and this idea is increasingly applied to coach education to enhance sport-oriented outcomes (40, 41).

However, the perspective of privatization from a normative standpoint has weak empirical support in sport-oriented contexts. Numerous large-scale synthesis studies conclude that privatization occurs through several different political means rather than through a universal efficiency model (42). Empirical research on voucher systems and subsidised private provision found that competition often result in increased segregation and stratification, therefore competitive activities do not validate the assertion of an expected universal quality improvement (43, 44). Thus, a core contradiction exists whereby, while privatization is perceived as enhancing equity, the frequent empirical finding of privatization exhibits differentiated access to benefits.

There are various methods of privatization that may affect the field of coaching. Although there is an analytical distinction between exogenous (e.g., outsourcing) and endogenous (e.g., manager-managed public systems) modes of privatization (23), this distinction has not been thoroughly outlined within sport and coach education literature. Research conducted within public education reveals that these distinct forms of privatization create uneven results that depend on a variety of factors. For example, procurement-based reforms could potentially create more competition within coaching but will be very sensitive to the capacity of the local governing body in determining whether this will occur (79). Another example can be found in shadow education market systems, which will create access for some learners, but limit access for others (45). While there are some studies identifying the presence of these distinct modes of privatization, the literature does not frequently compare the impact of these modes.

#### 2.2.2 Equity, stratification, and governance

A number of studies have cast doubt on whether privatization has real benefits globally and highlighted the issues of governance and equity that are often central to privatization debates. A great deal of research shows that privatized systems tend to create segmented access, providing access to wealthy and urban populations at the expense of public provision (46–48). While there is some evidence to suggest that regulatory interventions can improve access for low-income households, these cases tend to be few and are highly context-specific (43), leading to further challenges with stability in equity claims. The issue of governance complicates matters further. Privatization creates asymmetries of information, gaps in regulation, and coordination challenges, particularly for systems with both public and private actors that operate in a networked fashion (41, 49). Importantly, research into procurement and policy networks has shown that the way in which meso-level implementation occurs—rather than the design of a policy—will often impact outcomes (79). However, a good deal of this research has been conceptual or case-based in nature, with little evidence to support it empirically from within sports systems such as Oman, thus creating an important contextual gap.

#### 2.2.3 Coach education in a privatized space

The empirical studies have shown inequities within coaching education systems via finance constraints limiting certification access for lower-income coaches, cultural biases towards dominant sports while marginalising women's and community sports, and lack of access to governance structures for key stakeholders (50). However, much of the evidence regarding these inequities has come from small-scale studies, thus leaving uncertainty regarding the extent to which these inequities exist. In addition to being under-resourced or fragmented, informal modes of learning (experiential) are becoming predominant, thus replicating exclusionary practices (51). This reflects a shift towards privatization, whereby the uplift of risk and costs has fallen to the individual and have been promoted through value justification through examples of choice.

The democratic and co-constructed coaches as an alternative are shown to have higher relevance, engagement, and practice change (17, 33, 52). However, these models are usually reliant on; public funding, inclusion, and free entry, which conflict with the commodification of coaches and systems of charging a fee. This reveals a fundamental inconsistency with the way that equity and critical development is practiced. Another complication to the sustainability of private systems is that while the system creates some efficiencies; it also produces additional multiple dependent on market demand, profit equals and decreased public funding therefore increasing systemic instability. Furthermore, design concepts have been proposed which include; transparency, equity controls and equitable public investment (53). However, these concepts continue to exist primarily as a normative definition that has been poorly tested and very limited evidence has been collected related to these principles in hybrid and evolving systems.

In theory, privatized providers could be an attractive alternate option to rigid non-proprietary formal systems due to their adaptability, i.e., the development of tailored programmes with mentoring and online support which are aligned with the contextual, democratic and social justice models of efficacious learning, according to evidence presented by (1, 26, 34–36, 54). The privatization might be able to develop solutions for many longstanding complaints regarding the traditional coach educator model, including: (i) de-contextualized content, (ii) lack of continuous support, and (iii) minimal engagement of coaches as active, thoughtful practitioners.

Although privatizing coach education has been presented as a way to increase efficiency, expand provision of coach education, and enhance delivery to the community in systems that are currently underdeveloped, evidence suggests that privatization does not necessarily lead to positive outcomes. The empirical studies consistently demonstrate contradictory findings and significant risk factors as a result of privatization. Specific risk factors are attributed to inequitable interpretations of policy success between governments and front-line stakeholders, concerns pertaining to access and equity, gaps in regulatory compliance, and issues regarding job security and overall workload (47, 55, 56). The privatization does not, in all cases, automatically resolve inherent systemic weaknesses. Rather, it has the potential to replicate and/or exacerbate existing inequities, with developing governance structures presenting even larger concerns.

Therefore, in fact, privatization could perpetuate the known issues of existing systems (high cost, non-flexible delivery and standardized “one-size-fits-all” curricula), which have been shown to produce poor instructional outcomes, inequitable access and a limited, strictly technical, performance-based focus (31, 52, 54, 57–59) thus, if market-based providers replicate the problems associated with existing coach education delivery systems and leverage their similarity for economic gain, not only will no improvement in pedagogical quality occur, but also no increase in equity.

From the literature reviewed, there is consistent evidence of multi-level tensions associated with coach development today. First, at the system level, privatization is generally rationalized based on goals of improved efficiency and innovation, yet frequently leads to unintended consequences (e.g., increasing stratification and complexity of governance). Second, at the coach education level, traditional dominant pedagogies reproduce the same inequitable structures as would be found in the marketplace, while more inclusive/critical alternatives are in most cases underrepresented. Lastly, there are still several significant gaps to be addressed with respect to coach education within under-researched countries (e.g., Oman).

### 2.3 Coach education in Oman

Oman's coach education system is undeveloped and institutionally fragmented, any attempt to move toward privatization will be far more of a high-risk policy intervention than a neutral reform choice. Therefore, a critical review aims to link structural deficiencies with larger debates about privatization, equity and governance issues and to demonstrate why specific empirical evidence from the relevant context needs to be presented before any privatized solutions will be supported. The lack of a nationally recognized coach education system is one of the most significant constraints on sport development in Oman (60). Current provisions are *ad hoc* and are governed in a weak manner. They are characterized by a plethora of non-coordinated coaching programs and have no standardized curriculum and no formal quality assurance requirements to validate the coaching programs being offered. While previous research has suggested developing a national accreditation system within both the context of best practices of coach education internationally, this suggestion is yet to be implemented (60, 61). Research conducted at higher education institutions indicates that the context and the connections between people will greatly impact how the coaching and learning process is perceived (62–64). Oman is at a disadvantage in comparison with other countries. While the coach education systems of other countries continue to have issues with effectiveness, pedagogy, and impact, they are all built on formally established frameworks and pathways for certification (10, 28, 80).

In Oman, coaches advance along inconsistent pathways, and as a consequence, their competencies are inconsistent. More broadly, some of the main challenges found in the literature include: (1) not having a full common structure or governance, (2) formally training coaches through an education programme, though often highlighted as not contextualised and low impact; and (3) governance tensions. These challenges exist in many other places as well, but there are greater implications in Oman because it is currently without a stable institutional foundation. One major shortcoming of existing research is that there are few empirical research studies that have examined the perceptions of stakeholders on privatization of sport in non-western nations. While the existing literature indicates that stakeholder perspectives are important for shaping policy outcomes, very few empirical studies of stakeholder perspectives from the Gulf Cooperation Council (GCC) countries including Oman have been conducted. The stakeholder perspectives of coaches and administrators are particularly important to the legitimacy, implementation, and effectiveness of any reform. This research addresses those gaps through context-specific, empirical investigation of coach education in the country of Oman.

There are four primary arguments supporting this study. Firstly, Oman's coach development system has serious structural flaws; there is a lack of institutional cohesion required to facilitate continuous, high-quality development pathways. Secondly, empirical data from around the world illustrate that even well-established systems of coach development are unable to demonstrate efficacy, indicating that neither structural nor pedagogical issues can be addressed through market approaches alone. Thirdly, there is a debate on the effectiveness of privatization in both the education sector and the sport sector; in particular, mixed results have been produced through privatization. There is an urgent need for local data as empirical studies continue to lack insight into how coaches in the GCC region understand privatization and quality assurance within the coach education system.

## 3 Research method

The objective of this research is to strengthen the sport sector in Oman. We aim to evaluate the coach education program, determining the needs of stakeholders, and suggesting evidence-based improvement strategies. The methodological approach involves an exploratory cross-sectional case study using quantitative methods. The methodology includes: research design, survey questionnaire development and validation, ethical consideration, development of data collection protocols, and data analysis.

### 3.1 Survey design and research question development

We developed the survey questionnaire based on the brainstorming with the research group and an extensive review of the literature. The questions in the questionnaire were developed in both Arabic and English, so that the maximum number of people could respond. The survey consisted of three types of questions; Open-ended, Multiple-Choice, and a Likert Scale (1 = Likely to trust, 2 = Uncertain of trustworthiness, 3 = Unlikely to trust). These categories helped in collecting both quantifiable and non-quantifiable data. The sections in the survey include; demographic information (nationality, gender, professional title, employment type, experience level, educational level, etc.), privatization and the perceived benefits. The study seeks to answer the main research question- How effective is the current coach education program in Oman in supporting sports coaching development? Following are the sub- research questions.

1. How the perceptions towards coach education effectiveness, barriers, and privatization are influenced by variables such as job title and experience.
2. What are the barriers to coach education programs in Oman?
3. To what extent privatization is needed and what benefits does it offer to coach education?
4. What revenue-generation mechanisms can support the sustainability of a coach education program?

### 3.2 Data collection

The survey was distributed among stakeholders in Oman, The targeted stakeholders include, Sports coaches, Sports officials, Athletes, and Physical education teachers (PE) (Appendix A). This helped us to obtain diversity in data with varying experience level, affiliations, etc. We used the convenience sampling for the data collection since our research team members were from both academics and sports organizations with good networking among sports stakeholders. To improve the reliability and validity of the survey a pilot test was conducted on 10 participants. The feedback proved useful in improving the survey structure and wordings. The data collection was done between 10th September and 15th November 2024. A total of 159 samples were collected, however 149 samples were used in the final analysis after cleaning and removing the duplicates.

The study was conducted following the ethical guidelines set by our institution. Before being invited to participate, all participants were made aware of the research's purpose and their own role in the research project. They were assured that their identity would remain confidential and that the data provided would also be kept confidential. These ethical considerations were designed to promote maximum honesty and authenticity in participants’ responses to ensure that response bias was reduced as much as possible.

### 3.3 Preliminary analysis

The demographic information and professional experience of participants was captured in the preliminary findings from the survey. The majority of respondents were male athletes (85%), followed by PE teachers (6%), Sports administrators (5%), and Sports coaches (4%). Most of the respondents had no formal training in coaching and the majority of the coaches volunteered to coach athletes. A large number of coaches were newly trained and had less than three years of experience as a coach. Substantial differences in perceptions related to the privatization of coach education were identified between athlete groups and coaching groups. Sports administrators provided the highest levels of support for privatizing coach education (*M* = 1.71, SD = 0.44), representing positive perceptions of privatization across all participants in these two groups. Sports coaches had a moderate degree of support for privatizing coach education (*M* = 1.52, SD = 0.50. Athletes provided a mixed response on privatization (*M* = 1.10, SD = 0.68), while PE teachers exhibited the lowest level of support for privatization (*M* = 0.80, SD = 0.58). The overall Cronbach's alpha was 0.78. Table 1 shows the demographic profile of respondents.

**Table 1:** Demographic profile of respondents.

Job experience	Percentage	Job position	Percentage
16–19 years	3%	Sports coach	2.7%
8–11 years	8%	Sports admin	10.8%
12–15 years	11%	PE teacher	13.5%
4–7 years	14%	Athlete	73.0%
Less than 3 years	65%		

Job type	Percentage	Current salary	Percentage
Part time	3%	350–700 OMR	5.4%
Full time	32%	701–1,051 OMR	8.1%
Volunteer	65%	Less than 350 OMR	86.5%

Educational qualification	Percentage	Place of formal training in sports coaching	Percentage
Diploma in Physical Education	2.7%	GCC	5.4%
Secondary education/General diploma	2.7%	Outside GCC	10.8%
PhD in Physical Education	2.7%	Oman	18.9%
Master in Physical Education	10.8%	No formal training in sports training	64.9%
Bachelor in Physical Education	21.6%		
Teaching qualification (Not in physical education)	59.5%		

Furthermore, the gender analysis indicated a large gap between males and females with respect to employment and job positions. The majority of male (≈68%) belonged to the player/athlete category while PE teachers were mainly female (≈10%). All genders were equally underrepresented in the positions of Sports coach and Sports administrator. The findings show clear gender stratification by job title. Moreover, there were significant differences in terms of educational qualifications based on gender. Male were more likely to have a “Teaching qualification (Not in PE)” than female, with over 50% of males holding this qualification. Females overwhelmingly had “Bachelor's in PE,” which represented about 8% of female respondents. The “Secondary education/General diploma” represented the fewest qualifications for all genders, with each below 5%.

## 4 Analysis

### 4.1 Barriers and effectiveness of current coach education program

**Figure 1:**
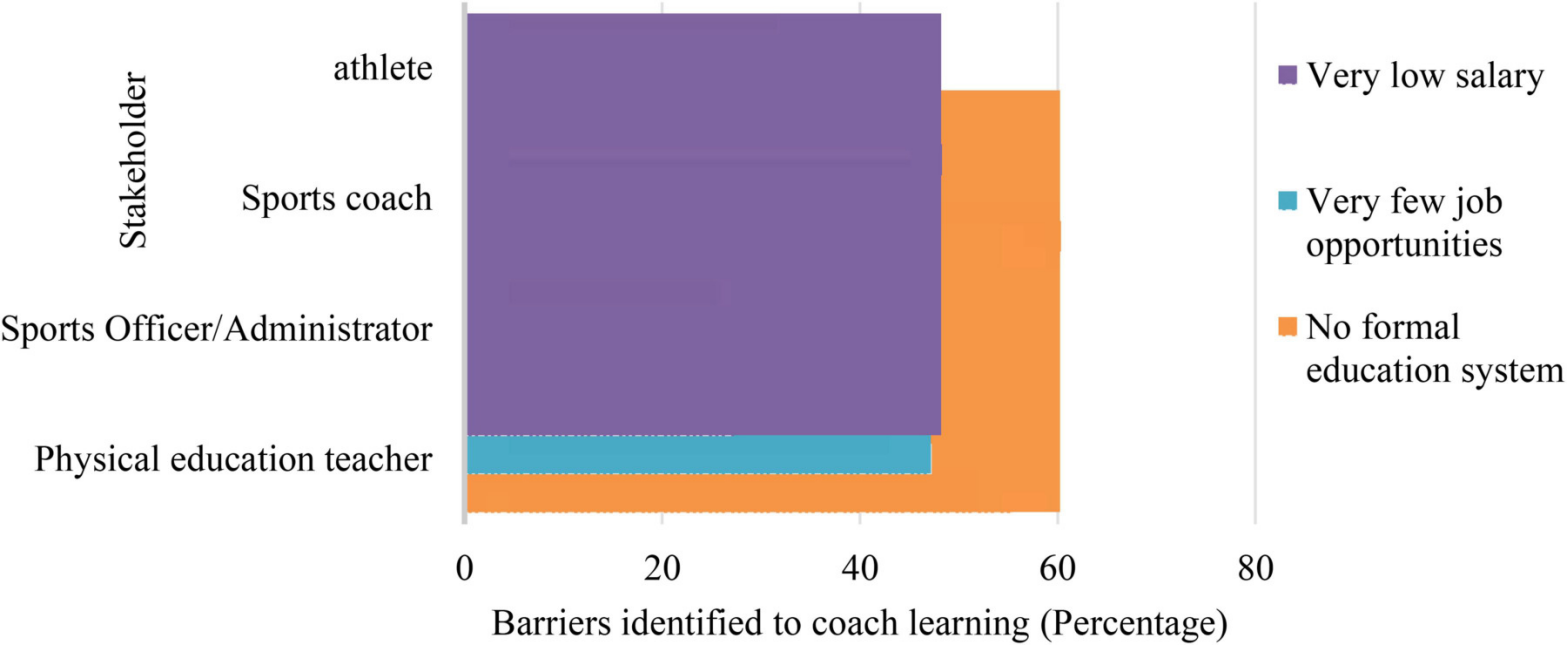
Barriers to coach education in Oman.

The PE teachers, Sports administrators, Sports coaches, and Athletes, indicated three primary obstacles to coaching in Oman, There is no formal coach education program, followed by limited coaching job opportunities, and low salary. However, the participants had slightly different perspectives towards issues based on their position within the system. The PE teachers were most concerned with the absence of a formal system (60%) followed by lack of available job opportunities (45%) and low pay (27%). The Sports administrators perceived job shortage as the main obstacle (43%) followed by absence of formal coach education programs (29%) and low pay (29%) each. On the other hand the Sports coaches showed equal concerns to each of the three barriers (33% for each). Athletes saw the absence of a formal coach education program (35%) and low salary (35%) as the main barrier followed by job shortage (30%). Figure 1 shows the barriers to coach education in Oman.

**Figure 2:**
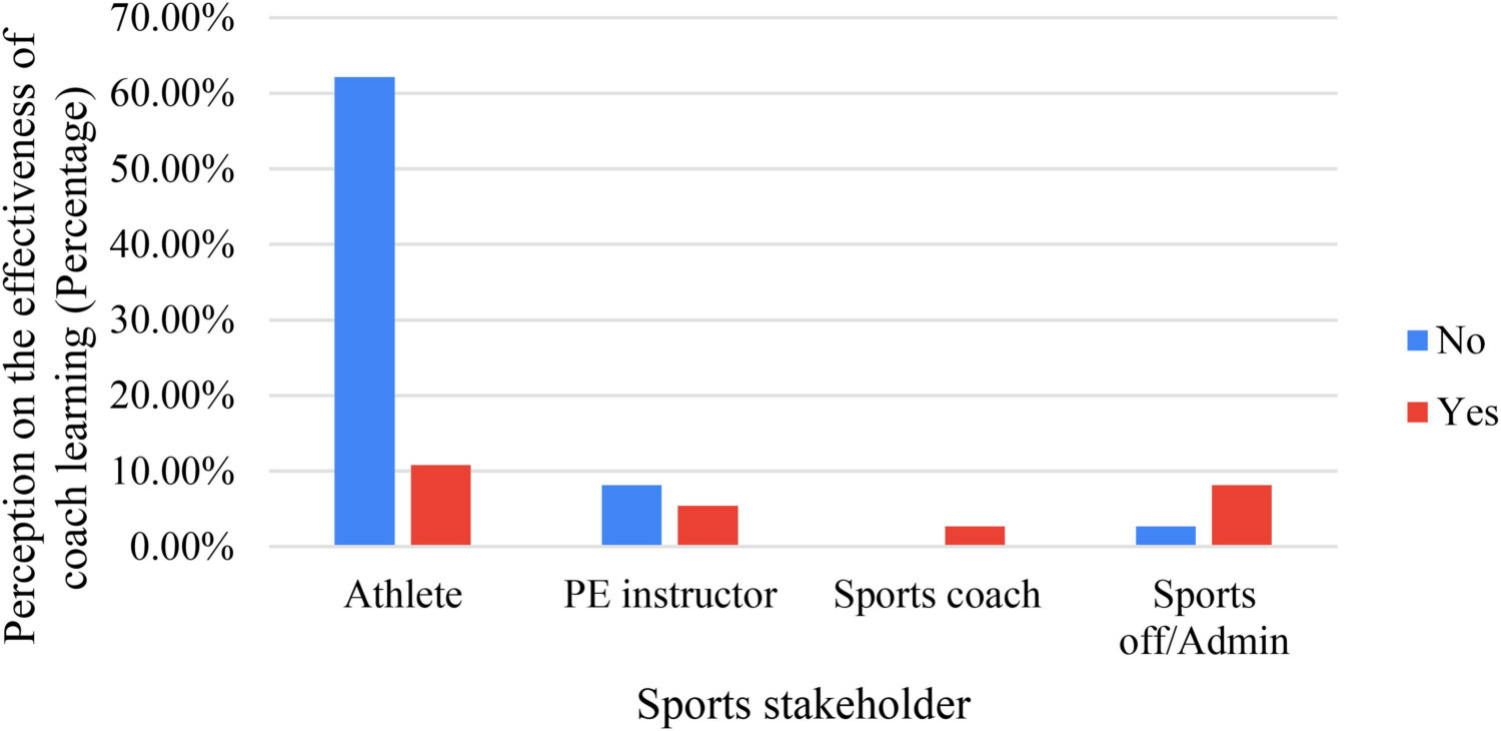
Stakeholders Perceptions on the effectiveness of coach education.

Additionally, the effectiveness of the existing coach education program was enquired. The majority of the respondents (70%) found the current coach education program is ineffective in meeting the sports need. Additionally, the Athlete profession accounted for the most of the negative response towards the current coach education program followed by the PE teachers. On the other hand the majority of the Sport administrators and Sports coaches believed that the existing coach education program is effective in meeting the sports need. There may be some biasedness due to their professions. Figure 2 shows stakeholders perceptions towards effectiveness of coach education.

### 4.2 Privatization of coach education

**Figure 3:**
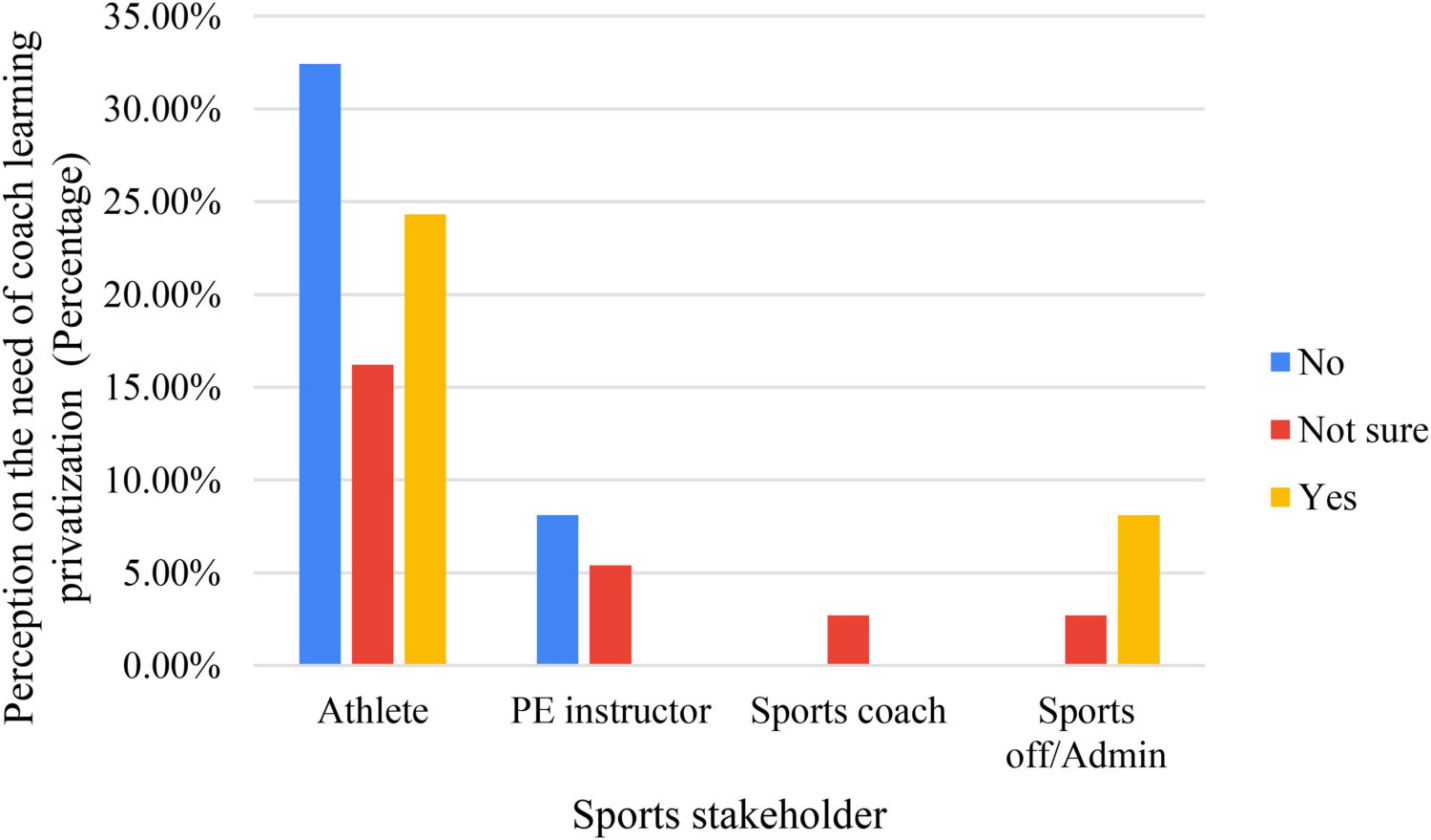
Stakeholder perception towards coach education privatization.

A detailed examination on the advantages of coach education privatization yielded a variety of responses. The PE teachers, Sports administrators, Sports coaches, and Athletes—expressed a strong favour towards privatization. A large percentage of respondents (75.7%) felt the need for privatization followed by 13.5% (somewhat necessary), and 10.8% (not needed). A cross examination based on the job title revealed considerable differences among respondents reporting the need for privatization. Athletes had the most respective support and opposition to privatizing coach education. Over 30% of them stated that they do not want privatization as compared to 24% (Yes). About 8% of PE teachers did not agree with the coach education privatization. The response from Sports coaches and Sports administrators indicated a more consistent willingness to support privatization than those of the other professional groups surveyed. While the non-support of privatization among all professional categories studied was highly apparent among Athletes, the support for privatization was fairly equally dispersed among all of the professions surveyed. Figure 3 shows the stakeholders perceptions towards coach education privatization.

Mann–Whitney *U*-tests were used for pairwise comparison in order to determine if there were any perceived differences in relation to the privatization of coach education across different stakeholders. Statistically significant differences were found in two groups. Between Sports administrators and Athletes a statistically significant difference noted in terms of their perceptions. The Sports administrators were significantly more supportive to privatization than Athletes (*p* = 0.015). Moreover, between Sports coaches and PE teachers, the PE teachers were significantly less supportive of privatization than Sports coaches (*p* = 0.008). Thus the significant differences in support for privatization based on the job title indicate the need for differentiation of policy and implementation. Table 2 shows the pairwise comparisons applying Mann–Whitney *U*-test.

**Table 2:** Pairwise comparisons applying Mann–Whitney *U*-test.

Variables	Mann–Whitney U test	Significance level (p)
Sports administrator vs. Sports coach	1,474	0.08
Sports administrator vs. athlete	1,562	0.01
Sports administrator vs. PE teacher	1,834	0.00
Sports coach vs. Athlete	1,340	0.50
Sports coach vs. PE teacher	1,608	0.01
Athlete vs. PE teacher	1,514	0.05

**Figure 4:**
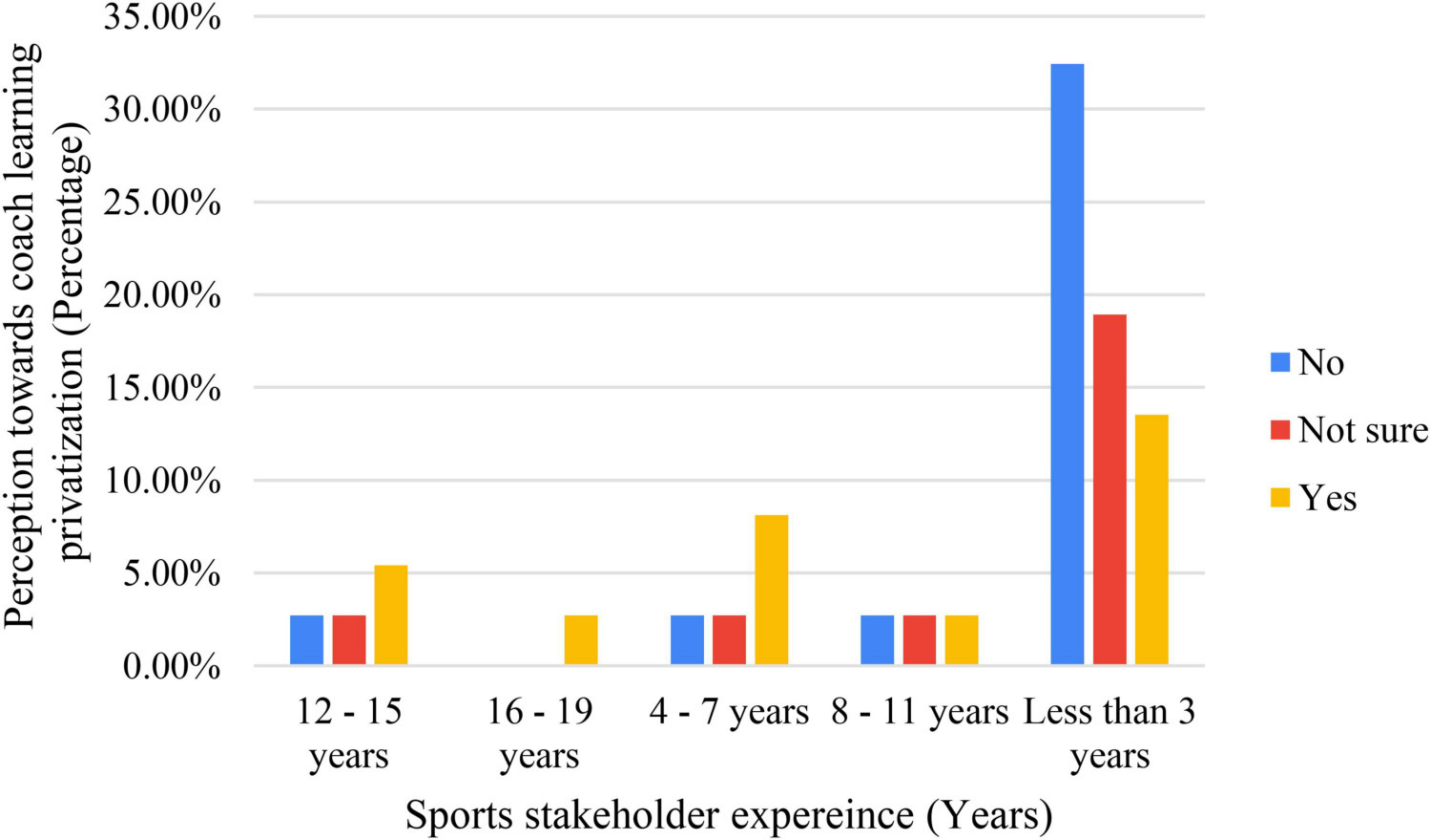
Experienced stakeholder perceptions towards coach education privatization.

The job experience-based analysis demonstrates a specific pattern towards coach education privatization. The analysis of job experience reinforces negative views towards privatization across respondents with less than three years of job experience (roughly 30% opposed). The respondent in this group exhibited the highest level of uncertainty (nearly 20%) and agreement (approximately 13%), which illustrates how fragmented and unsettled their opinions are concerning privatization issues. On the other hand the experienced groups (4–7, 8–11, 12–15, and 16–19) showed some degree of consistency in response patterns (neutral or agreeing) than in opposing privatization. Also, the 4–7 years group had nearly the next most supportive level of support for public-private partnerships (about 8% agreed). Figure 4 shows experienced stakeholders’ perception towards coach education privatization.

### 4.3 Coach education privatization benefits

**Figure 5:**
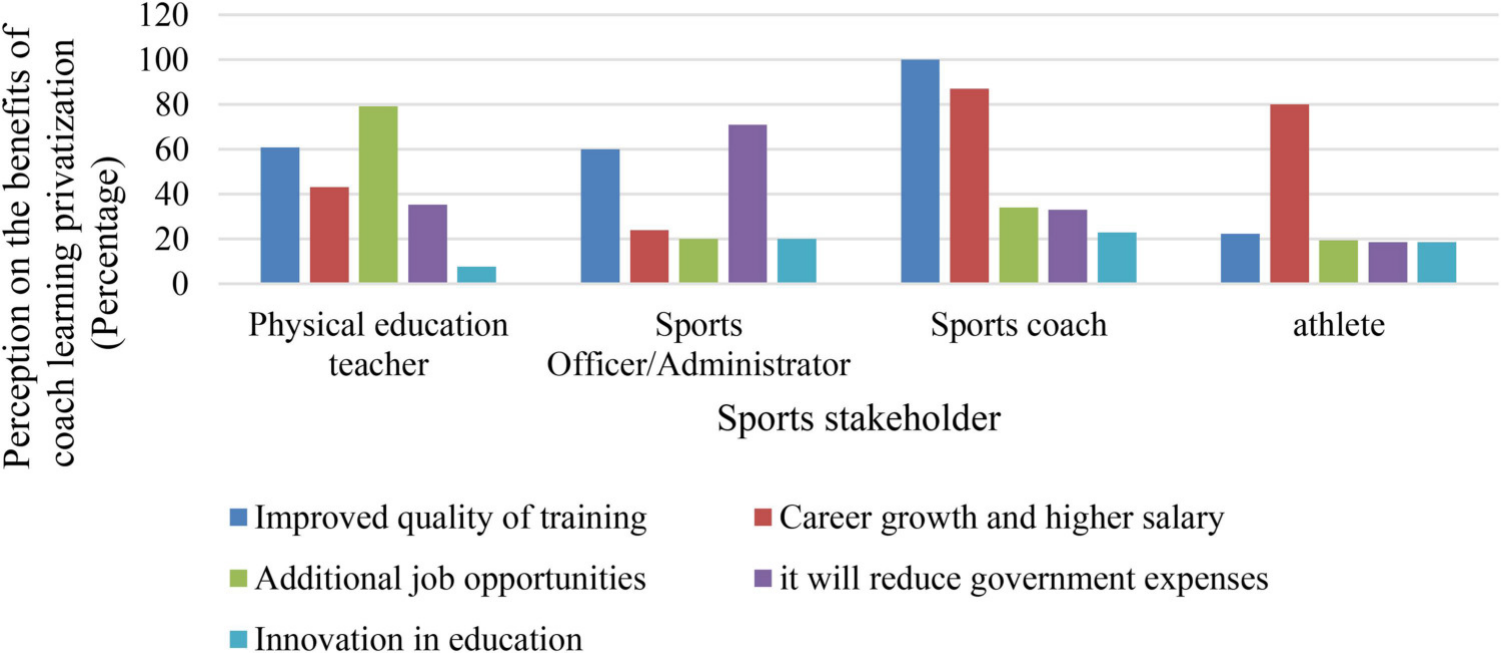
Sports stakeholders perceived benefits of coach education privatization.

A number of benefits of privatization were highlighted by the sports stakeholder. According to PE teachers privatization improves training quality “Improved quality of training” (30.77%) followed by “Career growth and higher salary” (23.08%) and “Additional job opportunities” (23.08%). In the case of Sports administrators, the most important factor was “Improved quality of training” (60%), followed by “Innovation in education” (20%). The most unified group was the Sports coaches with 100% of responses identifying benefits as “Improved quality of training.” The Athletes group believed the most important benefits being “Improved quality of training” (22.22%), “Career growth and higher salary” (21.30%), and “Additional job opportunities” (19.44%). This variation indicates that stakeholder expectations in the sport ecosystem varies according to their professions. Figure 5 shows sports stakeholders perceived benefits towards coach education privatization.

**Figure 6:**
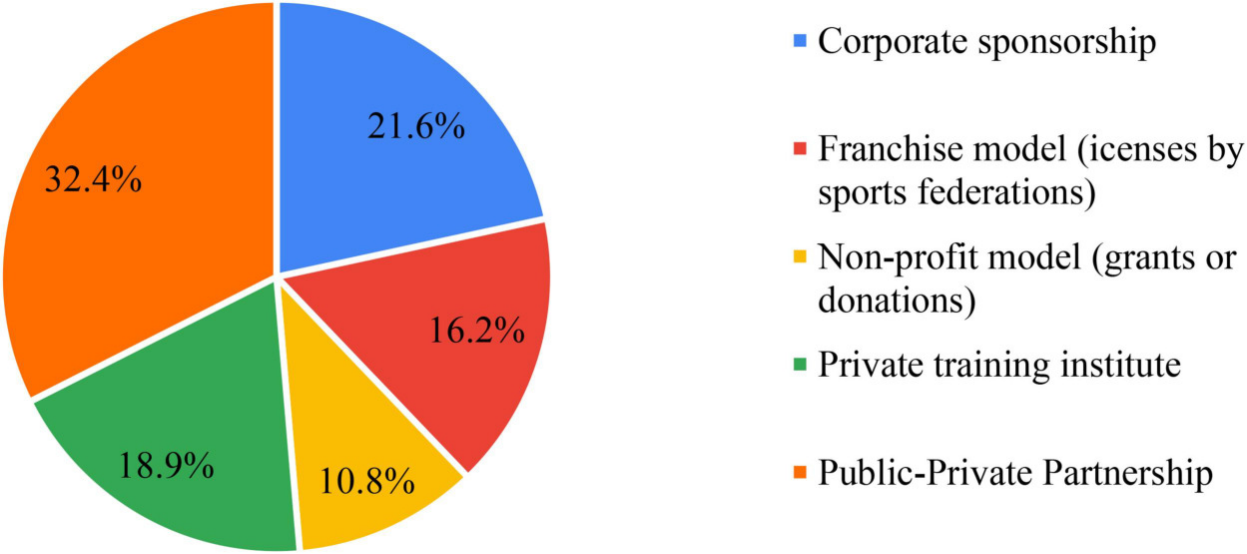
Effective privatization model for developing coach education in Oman.

Furthermore, the participants were enquired about the coach education development model supporting privatizing coach education in Oman. Their top most preference was Corporate Sponsorship (32.4%) followed by Franchise model (21.6%), Non-Profit model (18.9%), Private Training Institutes (16.2%), Public-Private Partnership (10.8%). The Franchise model includes sports federations issuing licenses, while the Non-Profit model relies on donations. Figure 6 shows the effective privatization model for coach education in Oman.

**Figure 7:**
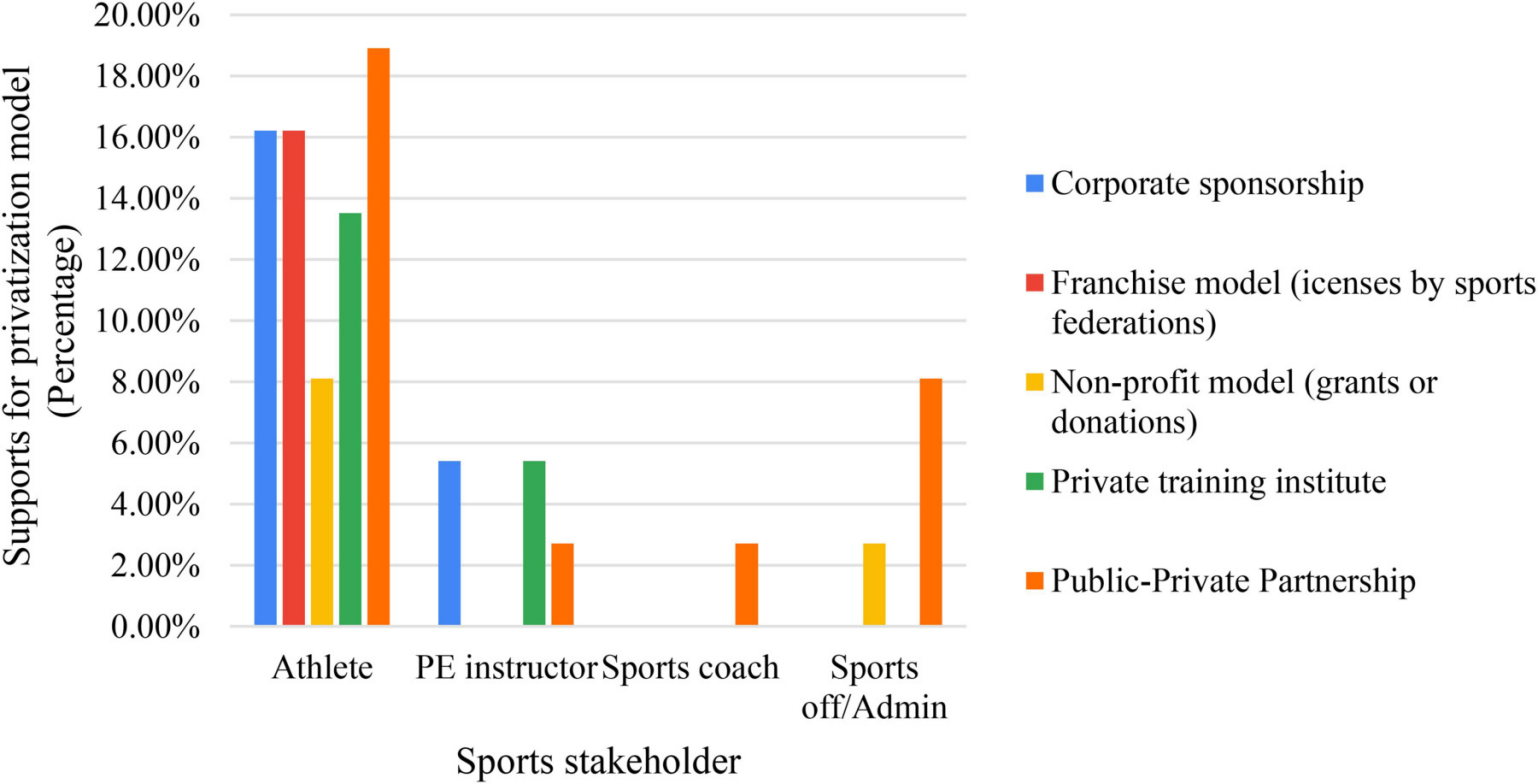
Support for privatization model in coach education development.

Additionally, support for various privatization models across job titles were enquired. The sports professional had different levels of support for the privatization models. The level of support from Athletes were highest for the Franchise model (more than 18%) followed by Corporate Sponsorship (approximately 16%). In contrast, the level of support shown by Sports administrators were highest for the Public-Private Partnership (approximately 8%), making it the preferred privatization option for this group. Figure 7 shows the stakeholders’ support for the privatization model in coach education development.

Lastly, Kruskal–Wallis *H* test was conducted to investigate differences in stakeholder preference towards privatization model. We found statistically significant differences for Private Sector Training [H (3, *N* = 149) = 8.63, *p* = .04] and for Non-Profit Model [H (3, *N* = 149) = 9.42, *p* = .03]. None of the other privatization models tested within this study expressed statistically significant differences in stakeholder preference. Therefore, the attitudes towards these two specific models are not uniformly distributed and are contingent upon professional affiliation. Table 3 shows stakeholder preference for the privatization model.

**Table 3:** Stakeholder preference for privatization model (kruskal–wallis *H*-test).

Variables	Test statistic (*χ*²)	Degree of freedom (df)	Significance level (*p*)	Effect size (*ε*²)
Public-Private Partnership	5.23	3	0.16	0.034
Corporate Sponsorship	7.49	3	0.06	0.048
Private Sector Training	8.63	3	0.04	0.054
Franchise model	4.42	3	0.22	0.029
Non-Profit model	9.42	3	0.03	0.050

### 4.4 Revenue generation methods through coach education

**Figure 8:**
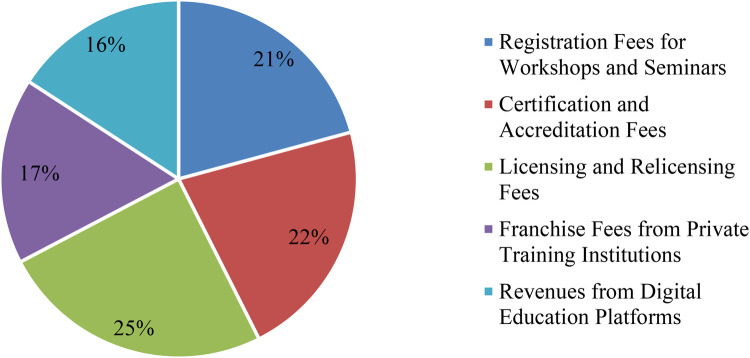
Revenue generation strategies in coach education.

We further investigated how the revenue can be generated from coach education. It was found that the digital education platform would contribute the maximum revenue. The 25% responded affirmation to the significance of digitally delivered systems of education followed by franchise fees from private training centers (22%). The other significant sources of revenue include registration fees for training/workshops/seminars (21%). Lastly coach registration/license fee (17%) and certification/accreditation fees (16%). Together these four types of funding can provide a balanced mix of revenues for coach education in Oman. Figure 8 shows the revenue generation strategies in coach education.

### 4.5 Kruskal–Wallis test for key variables and job titles/positions

We further analyzed variables such as the effectiveness of the current coach education program, coach education privatization, privatization attract investments, willingness to enroll in private coach education programs, and impact of combining learning methods (blended learning). The Kruskal–Wallis H test revealed statistically significant differences across the four stakeholder groups (Sports coaches, Athletes, Sports administrators, and PE teachers) for two key variables. First, perceptions of the effectiveness of current coach education programs differed significantly H (3) = 9.57, *p* = .023, indicating that at least one group evaluates the existing system differently from the others. Second, willingness to enroll in privatized coach education programs also varied significantly across groups H (3) = 8.40, *p* = .038, suggesting differences in acceptance or readiness regarding privatization initiatives.

Moreover, no statistically significant differences were found regarding the need for coach education privatization H (3) = 5.16, *p* = .161, nor for whether privatization would attract investments H (3) = 5.49, *p* = .139, suggesting shared views on these structural and financial considerations. Similarly, perceptions towards blended learning methods did not differ significantly across groups H (3) = 5.95, *p* = .114, indicating consistent agreement on the value of blended learning approaches. Table 4 shows the results of the overall Kruskal–Wallis test for each key variable.

**Table 4:** Krushkal wallis test for key variables.

Variable	H-statistic	df	*p*-value	Significance
Effectiveness of current coach education	9.57	3	.023	Significant (*p* < .05)
Need of coach education privatization	5.16	3	.161	Not significant
Will privatization attract investment	5.49	3	.139	Not significant
Willingness to enrol in a private coach education	8.40	3	.038	Significant (*p* < .05)
Impact of combining learning methods	5.95	3	.114	Not significant

Degrees of freedom (df) calculated as k—1, where *k* = 4 stakeholder groups.

Post-hoc pairwise comparisons were conducted applying Wilcoxon rank-sum tests with Dunn–Bonferroni test adjustment for variables that showed significant Kruskal–Wallis test results. For both the effectiveness of current coach education and willingness to enroll in private coaching programs, none of the pairwise comparisons remained statistically significant after adjustment (*p* > .05). Although some comparisons approached significance prior to adjustment, the conservative Bonferroni correction reduced statistical significance. These findings suggest that the observed overall differences are likely due to moderate variations across multiple groups rather than strong differences between specific pairs of stakeholders. Table 5 shows the Dunn's *post-hoc* pairwise comparisons of stakeholder groups.

**Table 5:** Dunn's *post-hoc* pairwise comparisons of stakeholder groups.

Variable	Group Comparison	*p* (Unadjusted)	*p* (Bonferroni)
Effectiveness of current coach education program	Athlete vs. PE teacher	.378	1.000
	Athlete vs. Sports coach	.155	.927
	Athlete vs. Sports admin	.056	.333
	PE teacher vs. Sports coach	.380	1.000
	PE teacher vs. Sports admin	.391	1.000
	Sports coach vs. Sports admin	.724	1.000
Willingness to enrol in private coach education program	Athlete vs. PE teacher	.186	1.000
	Athlete vs. Sports coach	.240	1.000
	Athlete vs. Sports admin	.068	.407
	PE teacher vs. Sports coach	.143	.859
	PE teacher vs. Sports admin	.020	.120
	Sports coach vs. Sports admin	.724	1.000

## 5 Discussion

This research confirms increasing amounts of evidence from around the globe that suggests access to coach education (i.e., through increasing either public or private facilities) and training through the use of market-based models will not automatically improve the quality. Thus raising a deeper question: What is missing in Oman is a coherent and contextually appropriate system of coach development that links learning pathways together and ensures the quality of coaching is regulated while providing coaches with development opportunities aligned to athlete needs. The literature supports this integration of formal, non-formal, and informal learning opportunities in coach education (30, 65), however, we found that this is not only undeveloped but also absent in Oman.

We have observed that there exists a heavy reliance on informal and self-directed forms of learning among coaches in Oman. Coaches use experiential processes to learn (e.g., through reflection, experimentation, peer interaction), thus supporting the assertion that learning by doing. This finding is consistent with prior studies (66, 81). Additionally, there is a paradox created by the use of informal learning methods. While informal learning provides flexibility and allows for contextual relevance, the informal learning takes place under no standard or validating process. Consequently, the question is not about the use of informal learning, but rather how informal learning will be incorporated into a structured coach education system.

The findings from the Kruskal–Wallis test showed statistically significant differences between stakeholders in terms of effectiveness and willingness to participate in the private coach education program, however, none of these differences were supported by *post-hoc* pairwise comparisons. Therefore, practically speaking, there are not clearly defined groups of stakeholders that are aligned around reform options, such as privatization, and that results demonstrate a fragmented perception of privatization that is characterized by dissatisfaction without cohesion. This is particularly important to consider in the context of privatization as it is frequently viewed as an answer to the inefficiency of current systems. The literature on privatization presents a conflict between market provision and equitable access, described by (23) as an unresolved trade-off. We also noticed that stakeholders’ views on reforms are significantly influenced by local and individual factors. Our findings mirror the study in Greece, where the perceptions of changes and evaluations varied based on institutional affiliation, experience, and educational level (67).

The privatization may represent a change in thinking about education as a good. This new way of thinking about education creates tension among those interested in improving efficiency through the use of privatization, those who are concerned about the equitable distribution of educational services, and those who are committed to long term educational development. While the use of private education may increase the flexibility and responsiveness of the education system, it also has the potential to create fragmented governance, decrease accountability, and produce differing quality standards. The critical literature has also pointed out that neoliberal economic and governance would prioritize controlling and standardizing over reflective opportunities for learning (17, 32, 52, 58, 68).

Another observation of this study is the revenue generation through multiple sources (licensing and certification, training and fees) by sports organizations in Oman. This indicates the willingness to embrace market-based strategies. The findings support a diversified financial model for coach education to reduce its dependence on government funding and ensure the system's long-term sustainability. But these approaches focus on fiscal sustainability rather than systemic coherence. Generating revenues does not resolve the fundamental issues with curriculum development, graduate outcomes, institutional fit, or pedagogical quality. Further, an over-emphasis on the financial model may restrict the intended purpose of coach education to economic outputs, thereby neglecting the developmental aspects of coach education.

Thus several concerns exist that a predominantly economic perspective on education reduces its purpose to merely creating a skilled labor force, which could undermine its broader social and human development objectives (40). The misalignment of these two fundamental purposes also creates tension within the context of Oman Vision 2040. While the Oman Vision 2040 focuses on increased levels of private sector participation, it also requires equitable access, social development and the building of national identity—objectives that do not automatically work together through market-based strategies. The importance of understanding the hidden implications related to the use of lifecycle costing in the context of privatization includes complexities related to regulating private enterprises, community reliance on private organizations, and the potential for instability in the system. As such, the priority should not be whether or not to privatize, but how to govern privatization while maintaining public value.

Moreover, the systemic barriers that prevent coaches from getting the best from their coach education program (e.g., the quality and relevancy) contains a plethora of issues. Formalised coaching courses are perceived by many coaches to be of very little benefit and not relevant to their coaching experiences (16, 31, 57, 59). Many coach education programs around the world have received much criticism because of their overly prescriptive and disconnected from the practice itself; even when done with adequate resources (27, 28, 80). This is particularly true in para sport (51). The concern here is that the issue lies not with the manner in which a service was provided, but rather the manner in which the system is currently designed or implemented (i.e., pedagogy). Therefore, without addressing issues that exist at these fundamental foundations of pedagogies, structural reforms, such as privatization, will not lead to significant changes particularly in countries like Oman.

The coaches around the world express a strong desire to have training delivered in ways that are relevant, interactive, affordable, and accessible (28–30, 69, 76, 82). The marketable products may be favoured at the expense of critical learning which could further inhibit the development of coaches through fostering the use of solid pedagogical practices, even though research shows us that there are currently barriers to supporting the development of both critical reflection and pedagogy by current coach education systems. Coach educators may continue to limit the development of innovative, critical thinking and changing pedagogical practices in their coaching programs by engaging in unthoughtful or conformist methods of teaching (16, 27, 37, 70).

The privatization from around the world reflects the complexity of the topic. In certain cases, private academies have become a new way of providing sport by innovating and filling in systemic gaps in the systems that they operate within while simultaneously increasing some of the factors that lead to commercialized pressures (10, 71, 72). The broader markets have demonstrated that the movement of coaching away from pedagogy to performance metrics and consumer expectations is a large problem for countries like Oman that have such a close association between sport and social development. However, there are also some benefits to the privatization process that should not be generalized into negative terms only. The private providers can allow for flexibility, innovation, and increased access, especially when using innovative forms of learning; however these advantages are dependent on having solid regulatory frameworks in place with established standards.

In general, it is anticipated that private companies will be able to offer coaches the flexibility necessary to provide coach education using new technologies–particularly online and digital technologies (10, 34, 73). Without the implementation of these protective measures, the privatization process will further increase inequity, decrease access, and concentrate more on programs that are commercially viable vs. those that are socially necessary. With the rise of digital and market-driven forces, the landscape for coach education is continuously being transformed. Additionally, AI-enabled platforms enable further access to coach education and facilitate the integration of digital literacy, data use, and technology competencies into coaching practice (73, 74). This indicates that context-sensitive systems, such as that of Oman, need to include digital and market innovation in ways beyond traditional public provision.

The stakeholders view current coach education in Oman as an underdeveloped and about 75.7% of the support reform. With reference to the frameworks of Verger et al. (42), Ball and Youdell (41), concerns arise regarding how market–oriented reforms, and accountability to the public, are not only perceived but also acted upon. The central issue in Oman relates to a system that lacks coherence. The privatization could provide flexibility (e.g., greater access to resources), there are also concerns regarding equitable access to services/equity, as well as the sustainability of these policies/systems when implemented as stand–alone solutions. Merely increasing availability of services—in either a public or private capacity—will do little to alleviate the problems created by fragmentation of coach education providers, weak governance of providers or limited pedagogical relevance of the educational experiences of coaches to mention. Therefore, what is needed is an approach which builds a coherent system, which aligns certification with practice, embeds learning in the local context and creates clearly defined developmental pathways for coaches. An alternative direction is to adopt a regulated hybrid model, whereby the public sector maintains responsibility for setting standards, providing accreditation and ensuring equitable access to coach education, while private and digital providers support the service delivery (including the flexibility and innovative practices). This approach avoids the false dichotomy of state vs. market as it recognizes the interdependence between governance, pedagogy and context in developing effective systems.

Therefore, it may be concluded that any improvements to the coach education system in Oman must involve system-level reforms rather than simply making changes to the structure of the system or destroying some aspect of it in order to provide more choice or competition. If privatization is pursued, it must be regulated and should, wherever possible, be consistent with the broader goals of the public sector. If not, the risk of creating superficial solutions that address non-existent efficiencies while continuing to leave significant structural issues unresolved or exacerbating inequities and fragmentation within the system will remain.

## 6 Conclusion

In this study we have examined the effectiveness of the current coach education ecosystem in Oman in supporting coaching development. The findings reveal that the existing system remains fragmented, under-resourced, and insufficiently aligned with international standards. Coach education provision is limited in scope and accessibility, resulting in a substantial proportion of coaches lacking formal qualifications and structured professional development pathways. In relation to roles, qualifications, and gender, the study identified structural inconsistencies between coaching responsibilities and certification requirements, alongside a clear gender imbalance within coaching environments.

The barriers such as limited career progression opportunities, comparatively low remuneration, and restricted access to continuous education further undermine professionalization. These challenges collectively hinder the contribution of coach education to broader national objectives, including employment generation and sustainable sectoral growth. The findings also highlight a strong stakeholder consensus regarding the need for reform through diversified and partially privatized delivery models. Public–Private Partnerships, corporate sponsorships, and market–oriented certification programs were discussed by participants as possible approaches for enhancing quality, expanding access, and supporting financial sustainability. These approaches can strengthen institutional accountability while improving responsiveness to labor market demands. Lastly, the stakeholders agreed on policy-related issues like privatization and investment potential, yet significantly diverged in evaluating the current system's effectiveness and their willingness to engage in privatized programs. This indicates structural consensus but varying assessments and readiness for change among sports stakeholders.

### 6.1 Theoretical and practical implications and recommendations

This research contributes to empirical evidence from an underexplored national context and offers a strategic roadmap for reforming the coach education program in Oman. The efficiency of coaches and coach education in Oman have been largely affected by both financial and non-financial constraints. This empirical study has added literature to the ever growing sport sector, however in the unexplored area of coach education in the context of the GCC region. It has presented the state and limitations of the current coach education program. Coach education in Oman requires the establishment of a national governing body responsible for curriculum development, accreditation, quality assurance, and financial sustainability. This will provide a strategic pathway toward professionalization, improved gender equity, and long-term sectoral sustainability.

The international partnerships between sports organizations, colleges and other academic institutions is very important to facilitate the sharing of knowledge and the professional development of coaches through collaborative projects. There needs to be greater alignment between academic institutions and sports organizations so that coaches can transition from theoretical learning to practical coaching via work placements or mentoring. In addition to these elements, developing a sustainable coach education model in Oman should rely on diversifying revenue sources, such as certification fees, paid training programs, digital learning platforms, corporate sponsorships, etc. This will decrease reliance on government funding while providing high-quality coach education and growth of sports in Oman.

Thus, this research does not presume that privatization is a desirable or a characteristic with regard to an outcome of goodwill. Instead, we studied the perceptions of stakeholders, a crucial step toward gaining insight into the circumstances, mechanisms, and implications of privatizing the development of coaches’ learning (i.e., whether or not to privatize coaching in Oman). Through the eyes of those within the local sports systems, this research provides further insight regarding the nature of current arrangements, thus leading to a better understanding of the particular realities of reform, specifically in developing sports systems where the globally based orientation towards market reform often clash with resource limitations, and culturally defined quality and accountability mechanisms.

Finally, the outcome of introducing private actors into a learning system that is lacking key foundational structures will not address the primary issues of quality, coherence, and access if the private actors systems are not viewed favourably by key stakeholders, negotiated effectively with key stakeholders, and implemented effectively with key stakeholders.

### 6.2 Limitations and future research

Several constraints and barriers were encountered while gathering the information on coach education including but not limited to a lack of reliable data, limited data available for certain demographic segments, as well as the inability to obtain adequate breadth of information from various domains or levels of coaching. Moreover, the inconsistency of results could have been impacted by differences in structure or perception of stakeholders towards coach education. Additionally, no secondary data exists for either private or public coaching education in Oman and therefore the research team was left to collect dispersed information through questionnaires surveys. Finally, the results of the study could be generalized however with caution due to exploratory nature. Overall, future research should move beyond descriptive studies toward theory-driven, mixed-method, and policy-oriented investigations that critically examine the effectiveness, equity, and sustainability of coach education reforms in Oman and comparable contexts.

## Data Availability

The raw data supporting the conclusions of this article will be made available by the authors, restricted by respondent confidentiality requirements.

## Appendix A

Table A1 Survey Instrument: A Study on the State of Coach education System in Oman.

**Table:**

Item No.	Section	Survey Item	Response Options
1	Demographic	Nationality	Omani; Non-Omani
		Gender	Male; Female
		Current role	Sports coach; Physical education teacher; Player/sportsperson; Sports administrator
		Employment status	Part-time; Full-time; Voluntary; Freelance
		Years of experience	Under 3 years; 4–7 years; 8–11 years; 12–15 years; 16–19 years; More than 20 years
		Monthly salary (OMR)	Under 349; 350–700; 701–1051; 1,052–1,400; 1,401–1,751; 1,752–2,000; Above 2000; No income
		Highest qualification in Physical Education	High school; Diploma; Bachelor's; Master's; PhD; Qualification in another field
		Level of team managed	Local; Regional; National; International
		Location of coaching training	Oman; GCC; Outside GCC; No formal training
		Source of training funding	Self-funded; Government-funded; Private sponsorship; No formal training
		Current coach education system effective?	Yes; No; Not sure
2	Privatization	Coach education should be privatized	Yes; No; Not sure
		Benefits of privatization (Select all that apply)	Improve quality; Career growth; Employment generation; Entrepreneurship; International collaboration; Innovation; Reduce government expenditure
		Most effective privatization model	Public–Private Partnership; Franchise model; Private academies; Corporate sponsorship; Non-profit model
		Government role (Select all that apply)	Regulation and monitoring; Accreditation; Curriculum design
		Government revenue sources (Select all that apply)	Licensing fees; Workshop fees; Certification fees; Franchise fees; Digital platform revenue
		Will privatization attract investment?	Likely; Unlikely; Not sure
		Likelihood of enrolling in private coaching program	Likely; Unlikely; Not sure
3	Learning and Development	Nature of current coach education	Formal; Non-formal; Informal; Experiential; Reflective
		Most effective learning method	Formal; Non-formal; Informal; Experiential; Reflective
		Effectiveness of blended learning	Likely; Unlikely; Not sure
		Importance of including Omani culture	Likely; Unlikely; Not sure
		Include Omani culture in programs?	Likely; Unlikely; Not sure
		Barriers faced (Select all that apply)	Lack of formal system; Limited job opportunities; Low salary; Other
4		Suggestions for improvement	Open-ended response

## References

[1] Li L Olson HO Tereschenko I Wang A McCleery J. Impact of coach education on coaching effectiveness in youth sport: a systematic review and meta-analysis. Int J Sports Sci Coach. (2024) 20(1):340–56. 10.1177/17479541241283442

[2] Marek P Krawczyński M Lenartowicz M. The role of the coach developer in the development of sports coaches’ competencies. J Kinesiol Exerc Sci. (2025) 109(35):31–40. 10.5604/01.3001.0054.8538

[3] Cheng Z Xu Y Zhou M Yin Z Xu L Chen W. Using the grounded theory to develop the theoretical model of Chinese school sports coaching competence. BMC Public Health. (2024a) 24(1):1–22. 10.1186/s12889-024-20649-9 39558322 PMC11571665

[4] Németh Z Shopulatov A Felder H Ridwan M Sobhkhiz A Basra MABA. Sport coach learning programs: perspectives and features. Revista Iberoamericana de Psicología del Ejercicio y el Deporte. (2024) 19(1):31–7. Available online at: https://www.riped-online.com/articles/sport-coach-education-programs-perspectives-and-features-105220.html

[5] Trudel P Milestetd M Culver DM. What the empirical studies on sport coach learning programs in higher education have to reveal: a review. Int Sport Coach J. (2020) 7(1):61–73. 10.1123/iscj.2019-0037

[6] Culver DM Werthner P Trudel P. Coach developers as ‘facilitators of learning’in a large-scale coach learning programme: one actor in a complex system. Int Sport Coach J. (2019) 6(3):296–306. 10.1123/iscj.2018-0081

[7] Mason RJ Farrow D Hattie JA. Sports coaches’ knowledge and beliefs about the provision, reception, and evaluation of verbal feedback. Front Psychol. (2020) 11(571552):1–10. 10.3389/fpsyg.2020.571552 33041941 PMC7522355

[8] Gano-Overway LA Dieffenbach K. Current practices in United States higher education coach learning programs. Int Sport Coach J. (2019) 6(2):226–33. 10.1123/iscj.2019-0013

[9] Hay P Dickens S Crudgington B Engstrom C. Exploring the potential of assessment efficacy in sports coaching. Int J Sports Sci Coach. (2012) 7(2):187–98. 10.1260/1747-9541.7.2.187

[10] Lara-Bercial S Bales J North J. Coaching around the world: on becoming a profession. In: Resende R Gomes AR, editors. Coaching for Human Development and Performance in Sports. Cham: Springer International Publishing (2021). p. 93–121.

[11] McCusker S Welply O. COACH: a cross-national study of coach training for teachers across 5 countries. Coach Int J Theory Res Pract. (2021) 14(1):39–61. 10.1080/17521882.2020.1735463

[12] Gök Y Aslan M. The comparison of the coaching education systems between countries: belgium, Germany, Estonia, France, Finland, Italy, Portugal, Spain, Switzerland, Türkiye and United Kingdom. Mediterr J Sport Sci. (2023) 6(2):374–405. 10.38021/asbid.1223071

[13] Borges M Rosado A Lobinger B Freitas F De Oliveira RF. Cultural intelligence in sport: an examination of football coaches’ cross-cultural training needs. Ger J Exerc Sport Res. (2023) 53(3):266–74. 10.1007/s12662-022-00825-y

[14] Cassidy T Jones RL Potrac P. Understanding Sports Coaching:The Pedagogical, Social and Cultural Foundations of Coaching Practice. 3rd ed. Abingdon: Routledge (2015). p. 1–63. 10.4324/9780203797952

[15] Urgun D Seidel J Vangeli E Borges M De Oliveira RF. Exploring the impact of cross-cultural training on cultural competence and cultural intelligence: a narrative systematic literature review. Front Psychol. (2025) 16(1511788):1–9. 10.3389/fpsyg.2025.1511788 PMC12009937 40260004

[16] Cushion CJ Griffiths M Armour K. Professional coach educators in-situ: a social analysis of practice. Sport Educ Soc. (2019) 24(5):533–46. 10.1080/13573322.2017.1411795

[17] Piggott D. The open society and coach learning: a philosophical agenda for policy reform and future sociological research. Phys Educ Sport Pedagogy. (2015) 20(3):283–98. 10.1080/17408989.2013.837435

[18] Vella SA Crowe TP Oades LG. Increasing the effectiveness of formal coach learning: evidence of a parallel process. Int J Sports Sci Coach. (2013) 8(2):417–30. 10.1260/1747-9541.8.2.417

[19] Bengtsson D Ivarsson A Stenling A Ntoumanis N Nygren J. Barriers and enablers of a formal coach development program: a multi-perspective process evaluation. Phys Educ Sport Pedagogy. (2025) 11:1–21. 10.1080/17408989.20252587028

[20] Martens R Vealey RS. Successful Coaching. 5th ed. Champaign (IL): Human Kinetics (2023).

[21] Grant AM. Steps to solutions: a process for putting solution-focused coaching principles into practice. In: Tee D Passmore J, editors. Coaching Practiced: Coaching Psychology Tools, Techniques, and Evidence-Based Approaches for Coaches. Hoboken (NJ): John Wiley & Sons, Inc (2022). p. 299–310. 10.1002/9781119835714

[22] Bray M. Educational planning, privatization and neoliberalism: evolving dynamics and challenges for the common good: m. Bray. Int Rev Educ. (2025) 71(5):917–39. 10.1007/s11159-025-10174-1

[23] Santalova A Põder K. Introduction: global trends in education privatization: a comprehensive exploration of endogenous and exogenous approaches. In: Santalova A Põder K, editors. Privatization in and of Public Education. New York (NY): Oxford University Press (2024). p. 1–4. 10.1093/oso/9780197673508.003.0001

[24] Jang J So WY Cho N Shin M. The hierarchy of sustainable sports coaching competencies in Korea. Sustainability. (2024) 16(2):1–14. 10.3390/su16020718

[25] Larkin P Barkell J O'Connor D. The practice environment—how coaches may promote athlete learning. Front Sports Act Living. (2022) 4(957086):1–8. 10.3389/fspor.2022.957086 PMC9352883 35935064

[26] Moen F Olsen M Bjørkøy JA. Investigating possible effects from a one-year coach-education program. Sports. (2020) 9(1):3. 10.3390/sports9010003 33375240 PMC7823483

[27] Watts DW Cushion CJ Cale L. Exploring professional coach educators’ journeys and perceptions and understandings of learning. Sport Educ Soc. (2021) 27(5):632–46. 10.1080/13573322.2021.1887115

[28] Nelson L Cushion C Potrac P. Enhancing the provision of coach learning: the recommendations of UK coaching practitioners. Phys Educ Sport Pedagogy. (2013) 18(2):204–18. 10.1080/17408989.2011.649725

[29] Ciampolini V Milistetd M Rynne SB Brasil VZ do Nascimento JV. Research review on coaches’ perceptions regarding the teaching strategies experienced in coach education programs. Int J Sports Sci Coach. (2019) 14(2):216–28. 10.1177/1747954119833597

[30] Stoszkowski J Collins D. Sources, topics and use of knowledge by coaches. J Sports Sci. (2016) 34(9):794–802. 10.1080/02640414.2015.1072279 26222481

[31] Wang Z Casey A Cope E. Coach experiences of formal coach education developed by national governing bodies: a systematic review. Phys Educ Sport Pedagogy. (2023) 30(3):351–63. 10.1080/17408989.2023.2230235

[32] Dempsey NM Richardson DJ Cope E Cronin CJ. Creating and disseminating coach education policy: a case of formal coach education in grassroots football. Sport Educ Soc. (2020) 26(8):917–30. 10.1080/13573322.2020.1802711

[33] Cope E Cushion CJ Harvey S Partington M. Investigating the impact of a freirean informed coach learning programme. Phys Educ Sport Pedagogy. (2021) 26(1):65–78. 10.1080/17408989.2020.1800619

[34] Cope E Alsowayen M Hosawi S Turkistani A Cushion CJ. Exploring the impact of a coach development programme through the perspectives of Saudi Arabian sport coaches. Phys Educ Sport Pedagogy. (2024) 31(2):241–55. 10.1080/17408989.2024.2319063

[35] Kjær JB. The professionalization of sports coaching: a case study of a graduate soccer coaching education program. J Hosp, Leis Sport To Educ. (2019) 24:50–62. 10.1016/j.jhlste.2018.11.001

[36] Newman TJ Santos F Pierce S Collins K Mercier V. Coach education and coach development within a contemporary social justice society: implications for future research and potential pitfalls. Quest. (2022) 74(3):234–50. 10.1080/00336297.2022.2080082

[37] Tinning R. Ruminations on reflection and critical pedagogy in sport coaching. Sports Coach Rev. (2021) 11(1):87–107. 10.1080/21640629.2021.1984045

[38] Balan A. Neoliberalism, privatization and marketisation: the implications for legal education in England and Wales. Cogent Educ. (2023) 10(2):1–15. 10.1080/2331186x.2023.2284548

[39] Belfield CR Levin HM. Education Privatization: Causes, Consequences and Planning Implications. Paris, France: Unesco, International Institute for Educational Planning (2002). p. 79. Available online at: https://www.academia.edu/download/52568176/ Fund74.pdf (Accessed April 10, 2026).

[40] Edeji OC. Neo-liberalism, human capital theory and the right to education: economic interpretation of the purpose of education. Soc Sci Humanit Open. (2024) 9:1–11. 10.1016/j.ssaho.2023.100734

[41] Ball SJ Youdell D. Hidden privatization in public education. Education International. *Institute of Education. University of London* (2008).

[42] Verger A Fontdevila C Zancajo A. Multiple paths towards education privatization in a globalizing world: a cultural political economy review. J Educ Policy. (2017) 32(6):757–87. 10.1080/02680939.2017.1318453

[43] Zancajo A Verger A Fontdevila C. The instrumentation of public subsidies for private schools: different regulatory models with concurrent equity implications. Eur Educ Res J. (2021) 21(1):44–70. 10.1177/14749041211023339

[44] Whitty G Power S. Marketization and privatization in mass education systems. Int J Educ Dev. (2000) 20(2):93–107. 10.1016/s0738-0593(99)00061-9

[45] Sarkar N Sharma A. Regulating coaching centres: reform paradoxes and market conundrum. Economic Political Weekly. (2025) 37(37):21. 10.71279/epw.v60i37.45171

[46] Bennell P. An education revolution: the privatization of schooling in capital city conurbations in sub-saharan Africa. Int J Educ Dev. (2024) 105(2024):1–10. 10.1016/j.ijedudev.2024.102988

[47] Hussain JA. Fostering educational excellence and addressing neoliberal challenges: perspectives from principals and educators in arab private schools in Israel. Policy Futures Educ. (2024) 23(2):410–26. 10.1177/14782103241280572

[48] Inglis P. Critical approaches to private education in the Global South. Sociol Compass. (2023) 17(5):e13080. 10.1111/soc4.13080

[49] Kohe GZ Collison-Randall H. Sport, Education and Corporatisation: Spaces of Connection, Contestation and Creativity. New York (NY): Routledge (2019). 10.4324/9781351128865

[50] Hölscher D Bozalek V Gray M. The relevance of nancy Fraser for transformative social work education. In: Morley C Ablett P Noble C Cowden S, editors. The Routledge Handbook of Critical Pedagogies for Social Work. Abingdon (Oxon): Routledge (2023). p. 245–59. 10.4324/9781351002042-21

[51] Townsend RC Huntley TD Cushion CJ Culver D. Infusing disability into coach education and development: a critical review and agenda for change. Phys Educ Sport Pedagogy. (2021) 27(3):247–60. 10.1080/17408989.2021.1873932

[52] Chapman R Richardson D Cope E Cronin C. Learning from the past; a freirean analysis of FA coach education since 1967. Sport Education and Society. (2019) 25(6):681–97. 10.1080/13573322.2019.1654989

[53] Berner A. Examining “privatization” and protecting equal rights. Front Educ. (2025) 10:1–4. 10.3389/feduc.2025.1621331

[54] Lervold K Haugan JA Østerås MGO Moen F. Exploration of factors predicting sport Coaches’ perceived performance. Sports. (2025) 13(3):83. 10.3390/sports13030083 40137807 PMC11946564

[55] Kumar H Manoli AE Hodgkinson IR Downward P. Sport participation: from policy, through facilities, to users’ health, well-being, and social capital. Sport Manag Rev. (2018) 21(5):549–62. 10.1016/j.smr.2018.01.002

[56] Haider N Gul F Amjad F. Impact of privatization of government schools on quality and access to education: perceptions of stakeholders. Global Educ Studies Rev. (2024) 9(3):31–9. 10.31703/gesr.2024(ix-iii).04

[57] Selimi E Lascu A Serpiello F Woods CT. Exploring football coaches’ views on coach education, role, and practice design: an Australian perspective. PLoS One. (2023) 18(5):e0285871. 10.1371/journal.pone.0285871 37192185 PMC10187903

[58] Holdom T Nichol A Ives B. Recognising, addressing and supporting the challenging nature of community sport coaching work: potential ways forward for research and practice. Sports Coach Rev. (2024) 13(2):265–76. 10.1080/21640629.2024.2335432

[59] Langan E Blake C Lonsdale C. Systematic review of the effectiveness of interpersonal coach education interventions on athlete outcomes. Psychol Sport Exerc. (2013) 14(1):37–49. 10.1016/j.psychsport.2012.06.007

[60] AL Busafi M. Coach learning in Oman. J Coach Educ. (2013) 6(1):43–60. 10.1123/jce.6.1.43

[61] Al Droushi AR Henry I. Modernization of athletics in Oman: between global pressures and local dynamics. Int J Hist Sport. (2020) 37(sup1):3–25. 10.1080/09523367.2020.1734565

[62] Mathew P Hakrob AN. Coaching in a higher education institution in the Middle East: reflections on the obstacles and the way forward. Int J Evid Based Coach Mentor. (2022) 20(1):66–82. 10.24384/6zcb-sc80

[63] Hakro AN Mathew P. Coaching and mentoring in higher education institutions: a case study in Oman. Int J Mentor Coach Educ. (2020) 9(3):307–22. 10.1108/ijmce-05-2019-0060

[64] Lyle J. Getting it right for everyone: sport coaching and the adult participation domain. Sport J. (2020) 41(2):1–18. Available online at: https://thesportjournal.org/article/getting-it-right-for-everyone-sport-coaching-and-the-adult-participation-domain/.

[65] Mesquita I Ribeiro J Santos S Morgan K. Coach learning and coach learning: portuguese expert coaches’ perspective. Sport Psychol. (2014) 28(2):124–36. 10.1123/tsp.2011-0117

[66] Abraham A Collins D. Effective skill development: how should athletes’ skills be developed. In: Collins DJ Abbott A Richards H, editors. Performance Psychology: A Practitioner’s Guide. Edinburgh: Elsevier (2011). p. 207–29. 10.1016/B978-0-443-06734-1.00015-8

[67] Stafylidis A Vantarakis A Stafylidis C Karagkiozi I Stafylidis S. Physical education teachers as adults trainers and their perceptions of their evaluation on vocational education and training. Cent Eur J Sport Sci Med. (2024) 46(2):65–79. 10.18276/cej.2024.2-06

[68] St. John EP. Higher education in post-neoliberal times: building human capabilities in the emergent period of uncertainty. Educ Sci. (2023) 13(5):1–18. 10.3390/educsci13050500

[69] McCarthy L Roberts C-M. A project-led framework for coach development in English men’s professional football: a premier league case study. Int Sport Coach J. (2024) 11(3):446–56. 10.1123/iscj.2023-0015

[70] Nash C Ashford M Collins L. Expertise in coach development: the need for clarity. Behav Sci. (2023) 13(11):924. 10.3390/bs13110924 37998671 PMC10668957

[71] Primus RS. Out of the shadows into the limelight: the impact of commercialization on the Swedish men’s elite football coach from the 1960s until today. Sport Soc. (2024) 27(12):1938–57. 10.1080/17430437.2024.2411490

[72] Skille EÅ Strittmatter AM Stenling C Fahlén J. The professionalization of the role of the coach: transforming the last bastion of the scandinavian welfare-sport model. Sport Soc. (2024) 27(12):1994–2013. 10.1080/17430437.2024.2411779

[73] Passmore J Woodward W. Coaching education: wake up to the new digital and AI coaching revolution!. Int Coach Psychol Rev. (2023) 18(1):58–72. 10.53841/bpsicpr.2023.18.1.58

[74] Passmore J Evans-Krimme R. The future of coaching: a conceptual framework for the coaching sector from personal craft to scientific process and the implications for practice and research. Front Psychol. (2021) 12:1–8. 10.3389/fpsyg.2021.715228 PMC8631535 34858257

[75] AL Busafi M. Impacts of globalization on sport and coach learning. Ovidius Univ Ann Ser Phys Educ Sport Sci Movem Health. (2015) 10(2):23–28. Available online at: https://analefefs.ro/en/

[76] McCarthy L. Reframing assessment in coach education and development programmes. In: McCarthy L, editor. Sport Coach Education, Development, and Assessment: International Perspectives. New York: Routledge (2024). p. 62–74. 10.4324/9781003472438-5

[77] Maclean J Lorimer R. Are coach education programmes the most effective method for coach development? Int J Coach Sci. (2016) 10(2):71–88. Available online at: https://rke.abertay.ac.uk/en/publications/are-coach-education-programmes-the-most-effective-method-for-coac-2/

[78] Cushion CJ Stodter A Clarke NJ. ‘It’s an experiential thing': the discursive construction of learning in high-performance coach education. Sport Educ Soc. (2021) 27(7):844–61. 10.1080/13573322.2021.1924143

[79] Holmqvist M. Lesson study as a vehicle for improving SEND teachers’ teaching skills. Int J Lesson Learn Stud. (2020) 9(3):193–202. 10.1108/IJLLS-05-2020-0022

[80] Walton J Cushion C Stodter A Cope E. A systematic review of coach developers’ professional learning. Sports Coach Rev. (2024) 13:1–26. 10.1080/21640629.2024.2429271

[81] Morgan K Jones RL Gilbourne D Llewellyn D. Changing the face of coach education: using ethno-drama to depict lived realities. Phys Educ Sport Pedagog. (2013) 18(5):520–33. 10.1080/17408989.2012.690863

[82] Mills JP Clements K. Effective sports coaching: a systematic integrative review [preprint]. OSF Preprints. (2021):1–56. 10.31236/osf.io/yhj9g

